# Effects of individual practice on joint musical synchronization

**DOI:** 10.3389/fnhum.2024.1381232

**Published:** 2024-05-22

**Authors:** Polina Plitchenko, Valentin Bégel, Caroline Palmer

**Affiliations:** Department of Psychology, McGill University, Montreal, QC, Canada

**Keywords:** musical synchrony, social interaction, duet synchronization, practice effects, delaycoupling

## Abstract

Successful music-making requires precise sensorimotor synchronization, both in individual (solo) and joint (ensemble) social settings. We investigated how individual practice synchronizing with a temporally regular melody (Solo conditions) influences subsequent synchronization between two partners (Joint conditions). Musically trained adults practiced producing a melody by tapping on a keypad; each tap generated the next tone in the melody. First, the pairs synchronized their melody productions with their partner in a baseline Joint synchronization task. Then each partner separately synchronized their melody with a computer-generated recording of the partner’s melody in a Solo intervention condition that presented either Normal (temporally regular) auditory feedback or delayed feedback (by 30–70 ms) in occasional (25%) randomly placed tone positions. Then the pairs synchronized again with their partner in a Joint condition. Next, they performed the second Solo condition (normal or delayed auditory feedback) followed again by the Joint condition. Joint synchronization performance was modeled with a delay-coupled oscillator model to assess the coupling strength between partners. Absolute asynchronies in the Solo Intervention tasks were greater in the Delayed feedback condition than in the Normal feedback condition. Model estimates yielded larger coupling values between partners in Joint conditions that followed the Solo Normal feedback than the Solo Delayed feedback. Notably, the asynchronies were smaller in the Joint conditions than in the Solo conditions. These findings indicate that coupled interactions in settings of two or more performers can be improved by individual synchronization practice.

## 1 Introduction

A primary goal of musical ensembles is to create a synchronous performance with fellow musicians. However, the majority of musical practice is accomplished individually, prior to an ensemble performance. How do solitary practice conditions influence subsequent joint performance? Auditory-motor synchronization, the simultaneous production of sound with a perceived auditory stimulus, can play a vital role in the perceived pleasantness of music (Bégel et al., 2022). We focus here on musical synchrony among partners; synchrony among species members is found in many life forms (Moiseff and Copeland, 2010) that display a simultaneous production of action with sound (Strogatz and Stewart, 1993; Large et al., 2015). Social interaction among group members also influences musical synchrony. For example, comparisons of individuals who synchronized with a metronome or with a partner changed the temporal predictability and adaptation of the partner’s behavior (Dumas and Fairhurst, 2021). Social interaction in musical groups requires strong coordination and is often characterized by coupled physiological systems (Delius and Mueller, 2022). Fewer studies have examined how individual practice helps musicians improve their ability to synchronize with others. The current study addresses how individual synchronization practice influences joint performance in dyadic synchronization among musicians.

### 1.1 Learning effects in music performance

A few studies have examined the impact of types of musical practice on the development of music performance skills. Caramiaux et al. (2018) investigated effects of tempo variability and different learning regimens in non-musician participants’ improvement in timing and motor skills during piano performance. Non-musicians were trained with different types of piano practice and then performed novel finger sequences on a piano keyboard. The performers’ timing displayed greatest improvement when the tempo during learning matched the tempo during transfer to the novel melodies. Furthermore, the study found a carryover effect of previously acquired skills in transfer to novel melodies, regardless of the movement task complexity. Thus, Caramiaux et al. (2018)‘s finding suggest that musical practice can improve subsequent performance regardless of the mechanical difficulty; improvement was found from learning task to subsequent transfer task, whether the consistent tempo was fast or slow.

Stambaugh (2011) examined the impact of different practice conditions on musicians’ solo performance. Novice clarinetists learned novel melodies in blocked or random practice conditions. Participants in the random condition completed six trials of each of the 3 melodies in a random order on each day of the practice. Participants in the blocked condition performed a single melody for 18 trials on each day of the practice. Outcomes were judged by the clarinetists’ performance accuracy, tempo, temporal evenness and attitude. The assessment of learning effects was done on the last trials of the final day, while retention was evaluated 24 h after the last day. Differences between the blocked and random training were found only in the performance tempo. The group in the random condition showed a higher playing tempo during retention and maintained their accuracy, in contrast to the blocked group. This suggests that the random interleaving of practice can enhance subsequent performance.

Bégel et al. (2024) addressed the causal relationship between practice in dyadic musical synchronization and subsequent solo synchronization. Pairs of musician and non-musician participants took turns synchronizing their melody by tapping with a metronome (each tap generated the next melody tone). In the Joint Intervention conditions, participants attempted to synchronize their melodies simultaneously with their partner either with normal auditory feedback (normal feedback) or with randomly placed delayed feedback on 25% of melodic tones (delayed feedback). After each Intervention, the turn-taking condition of synchronizing with the metronome was repeated. Partners’ asynchronies with the metronome were larger following the delayed feedback performances than following the normal feedback performances. Furthermore, partners’ social interaction ratings of connectedness, relationship with their partner, and synchronization judgments were reduced after the delayed feedback condition relative to the normal feedback condition. These findings suggest that practice with a partner increases synchrony that carries over to subsequent individual performance. We ask the reverse question in this study: Does individual practice affect subsequent dyadic performance with a partner?

### 1.2 Learning and reliance on auditory feedback

A key factor in music performance training is a reduced reliance on auditory feedback to guide future actions. For example, musically trained individuals can perform familiar music accurately in the absence of auditory feedback (Finney, 1997; Repp, 1999), and beginners rely on that feedback during practice more so than advanced musicians (Luciani et al., 2022). Other studies have confirmed that auditory feedback is critical for initial learning of novel musical pieces but is relatively independent at retrieval, once learned (Finney and Palmer, 2003). One explanation for this is that auditory imagery can reduce a performer’s reliance on auditory feedback (Highben and Palmer, 2004; Bishop et al., 2014).

Group music-making forces a higher reliance on auditory feedback in order for musicians to perceive one’s own performance outcomes relative to those of other musicians. Interaction among musical group members requires them to distinguish sounds produced by oneself from those produced by others (Keller et al., 2016). Studies have addressed distinctions between self-other integration and self-other segregation in trained musicians (Liebermann-Jordanidis et al., 2021). We address here the extent to which individual practice that enables musicians to distinguish their own actions from a recording, via manipulations of auditory feedback, influence the subsequent balance of self-other integration and segregation in joint performance.

### 1.3 Synchronization models of joint performance

Musically trained individuals tend to show smaller asynchronies of their tone productions with a regular auditory stimulus than do untrained individuals (Repp and Su, 2013), suggesting that long-term practice on a musical instrument improves synchronization. Large et al. (2015) proposed that sensory and motor networks interact to improve musical synchrony and the overall timing as individuals gain performance experience. A sensory (auditory) network and a motor network were modeled with nonlinear oscillators whose coupling strength increased as a function of the periodic signals encountered in musical stimuli. Participants tapped along with the perceived pulse in the melodies presented. Large et al.’s (2015) model predicted the perception of a regular musical beat at specific frequencies, consistent with the empirical findings. The model captured the degree of coupling between auditory and motor neural oscillations that influenced the participants’ perception of a regular musical beat and synchronization. Musicians’ increased auditory-motor interactions contribute to improved memory for musical sequences as well (Palmer, 2005; Mathias et al., 2015).

Several findings suggest that human synchronization with a regular auditory cue, such as a metronome, tends to be anticipatory, called a negative mean asynchrony (Repp and Su, 2013). Anticipation refers to the tendency for participants’ tone onsets to occur earlier than the tones with which they intend to synchronize. The integration of information at a central level from different sensory modalities was proposed to account for the anticipatory nature of synchrony in single-subject auditory-motor studies (*cf.* Aschersleben and Prinz, 1995; Castro-Meneses and Sowman, 2018). Other explanations of anticipatory behavior have been proposed that distinguish among internal states (Palmer and Demos, 2022): the first, weak anticipation, suggests the creation of an internal model that helps an individual to make predictions of future events and act on those predictions (Clark, 1997). The second explanation is strong anticipation, which assumes a relationship between an external stimulus “driver” oscillation, such as an auditory metronome, and an internal “driven” oscillation, such as a human (Voss, 2000). In this theory, one of the oscillators creates the oscillation while the other oscillation follows that oscillation at a time delay, predicting anticipation via a time-delayed memory of the system’s previous state (Stepp, 2009; Stepp and Turvey, 2010; Demos et al., 2019). These models, called delay-coupled oscillator models, have been applied to musical ensembles (Demos et al., 2019; Palmer and Demos, 2022) and to other physical systems (Machado and Matias, 2020) to explain coupling relationships and their influence on synchronization.

Demos et al. (2019) tested a strong anticipation model fit to the synchronization of dyadic partners’ musical performances. A bidirectional delay-coupling model was applied to the partners’ asynchronies as their auditory feedback was manipulated. Equation 1 shows the delay-coupled model applied to two partners’ relative phase values (measure of tone onset asynchrony). The bidirectional coupling permits interaction and synchrony of two oscillators (partners) that receive and adapt to the auditory feedback from themselves and from their partner (Stepp and Turvey, 2010; Bégel et al., 2022). Three parameters include ω, the oscillator’s intrinsic frequency (represented as period, the inverse of rate); a coupling term *k* that influences the amount of adaptation of one oscillator to another; and a time delay *t* (ms) that represents the system’s memory for a past state [for full constraints on the model’s parameters, see Demos et al. (2019) Supplementary materials].


(1) $$\cdot \theta_{1}=\omega_{1}+k_{1}\left(\theta_{2}-\theta_{1,\tau 1}\right),\cdot \theta_{2}=\omega_{2}+k_{2}\left(\theta_{1}-\theta_{2,\tau 2}\right)$$

Demos et al. (2019) tested the model in musical duets by removing the auditory feedback matching one partner’s part (for example, the driven partner) from both partners to test the model’s ability to adapt to the leader role (the partner whose auditory feedback was not removed). Unidirectional coupling occurred when the partner whose feedback was removed (the “driven” partner) attempted to maintain their synchrony with the partner who is not able to adjust (the “driver” partner). Importantly, the driven partner showed anticipatory synchrony by performing slightly before the driver partner. The partners’ asynchronies were fit with the delay-coupled model whose findings showed a stronger coupling between partners when full feedback was present; a unidirectional coupling of the partner whose feedback was removed to the partner whose feedback was present; and no coupling when both partners’ feedback were removed. The degree of coupling corresponded to the size and directionality of the asynchronies among the partners across the auditory feedback conditions. Thus, this study illustrated that delay-coupled models of synchrony can address anticipatory behavior in terms of coupling between partners, without the need for an internal model. We apply the model here to examine how the unidirectional coupling experience offered in solo synchronization with a computer-generated recording can influence bidirectional coupling that typically occurs in joint (dyadic) synchronization.

Alternative models have proposed that synchronization in a musical ensemble relies on a process of mutual correction and adjustment among the performers that can be defined via linear error correction factors (Wing et al., 2014; Jacoby et al., 2015). An error correction model was applied to the synchronization measures from members of two string quartets (Wing et al., 2014). The linear phase correction model showed that the other performers exhibited stronger correction of their tone onsets’ relative phase values to follow the leading part (the first violin). Estimates of the correction gain were slightly below the 0.25 chance value for 4 performers, considered an optimal value for minimizing the asynchrony variability.

Some synchronization models are built on the assumption of internal models that generate predictions for a partner’s actions. Keller et al. (2007) measured pianists as they synchronized with a recording of their own performance or a partner’s performance recording; asynchronies were reduced in the condition in which they synchronized with their own performance relative to other performances. These findings were interpreted as support for an internal model for one’s own performance, compared with a less precise internal model for other pianists’ performances. Van der Steen and Keller (2013) proposed the adaptation and anticipation model (ADAM), that posits both adaptive and anticipatory behaviors of sensorimotor synchronization. The ADAM model has been extended to duet performance, but it has not tested effects of learning on subsequent performance or comparisons between solo and joint performance. Thus, there remains a gap in understanding how individual synchronization practice influences synchronization in joint music performance.

### 1.4 Current study

The focus of the present study was to investigate the impact of learning interventions in which musically trained individuals synchronized their melodies with a computer-generated auditory recording of a partner’s melody, on subsequent joint performance. Adult participants with musical training participated in Solo practice conditions in which they synchronized with a computer-generated recording, followed by Joint Performance conditions in which they synchronized with a partner. In one Solo Intervention condition, normal auditory feedback was presented, and in the second Solo Intervention condition, auditory feedback was occasionally delayed on 25% of randomly place tones; the order of the Solo Intervention conditions was counterbalanced across pairs. All participants performed both Solo interventions and in subsequent Joint performance conditions. Synchronization performance was measured during all Solo and Joint conditions. First, we predicted that synchronization in the Solo conditions would be worse for the occasional delayed auditory feedback than for the normal feedback. Second, we predicted that synchronization measures in the Joint performance conditions should improve more following the Solo intervention with normal feedback than delayed feedback. Third, we predicted greater coupling between partners in Joint synchronization conditions that followed normal auditory feedback than delayed feedback, based on a delay-coupled bidirectional model (Demos et al., 2019) applied to the partners’ asynchronies.

## 2 Methods

### 2.1 Participants

The participants were recruited from the Montreal community through Facebook and through the McGill community participant pool. All 50 participants (age range = 18–33 years, *M* = 22.3, SD = 3.4; 37 were female), referred to here as Musicians (years of training on a musical instrument = 6–16 years, *M* = 10.2, SD = 2.4) had at least 5 years of private instrumental musical training on their primary instrument in a classical Western style that includes the goal of synchronous tone onsets in joint performance. Participants were excluded if they exhibited hearing loss in a screening test or did not meet the study requirements to synchronize their taps with a regular metronome. Two additional participants were excluded due to data loss resulting from experimental error. An audiometry screening confirmed that participants had normal hearing in the frequency range of stimuli used in the experiment (<30 dB HL for single tones in the 125–750 Hz range). The study was approved by the McGill University Research Ethics Board for the duration of the research project.

### 2.2 Equipment and stimuli

Participants tapped an eight-note melody on a force-sensitive pad controlled with Arduino and connected to a Linux computer (Dell T3600 running Fedora 16) via MIDI. Participants heard the melodies through the headphones (AKG K240 Studio) in a marimba timbre (GM2, patch #13, channel #1;2; fixed velocity: 100) produced with a Roland Studio Canvas SD-50 tone generator and MOTU soundcard. Metronome beats were presented in a high-pitched woodblock timbre (GM2, patch #116, channel #4; fixed velocity: 127). Timing of the presented metronome sounds was controlled by the FTAP program (Finney, 2001). Delay between the tap onset on the force-sensitive pad to the tap being recorded, was less than 3 ms, which includes the signal passing through Arduino to M-Audio Uno MIDI device then into Linux internal delay and FTAP (Tranchant et al., 2022). The time delay from a finger tap on the Arduino pad to the start of the sound was less than 1.0 ms (Scheurich et al., 2018, Supplemental materials).

An ascending/descending melody in G major, composed of G4 – A4 – B4 – C5 – D5 – C5 – B4 – A4, was used for partner A’s higher-pitched feedback in all experimental conditions. The same melody in G major, one octave lower (G3 – A3 – B3 – C4 – D4 – C4 – B3 – A3) was used for partner B’s lower-pitched feedback in all experimental conditions. The G major melody was chosen for its familiarity and the pitch difference between the two melodies was created to ensure that each partner’s melody could be differentiated by the participants.

Stimuli in both the Joint and Solo synchronization conditions were based on synchronization-continuation trials. Each trial began with 8 beats of a metronome sounded every 450 ms (C6) with a woodblock timbre, to set the initial tempo. Participants started tapping on the 9th beat and the metronome stopped after another 8 beats while the participants continued to synchronize with their partners for 9.5 melody repetitions (76 taps) until the sound delivered to headphones ended, indicating the trial end. The data from each trial in all Solo and Joint performance conditions included the first 72 taps (9 repetitions) after the metronome ceased; the final 4 taps were excluded from analysis.

Additionally, stimuli in the Solo intervention conditions presented a computer-generated recording of one partner’s melody with either Normal auditory feedback (temporally regular tone onsets) or Delayed auditory feedback. The Delayed Auditory feedback occurred in 25% of the tones (distributed evenly among the two melodies) and ranged from 30 to 70 ms with a mean delay of 50 ms. The delay was pseudo-randomly positioned in the melodies to avoid the initial and final tones in each trial.

### 2.3 Design

**Figure 1:**
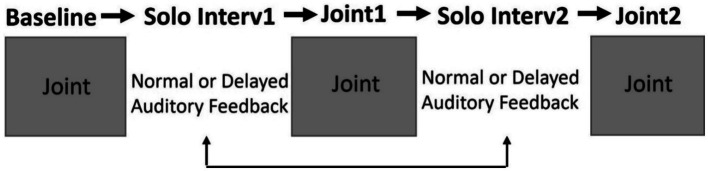
Order of the Solo Intervention conditions and the Joint synchrony conditions. Half of the partner dyads performed the Interventions in the two different orders.

Each pair of participants performed all tasks in this within-subject design. The experiment consisted of two different synchronization tasks: A Joint performance task and a Solo intervention task. The Joint performance task occurred three times and alternated with the Solo intervention task which occurred twice. The order of tasks is shown in Figure 1. The three Joint performance conditions are referred to Baseline Joint (initial condition), Post-Delay Joint (following the Delayed Auditory Feedback) and Post-Normal Joint (following the Normal Auditory Feedback). As shown in Figure 1, the conditions alternated in order to measure the impact of the interventions on the subsequent Joint performance.

The order in which the partners received the two Solo intervention conditions was counterbalanced across pairs. Additionally, the order in which the two partners assigned to upper or lower melody (A or B) performed the Solo intervention conditions was counterbalanced across pairs. Each of the Solo intervention and Joint performance conditions contained one practice trial and 3 experimental trials.

### 2.4 Procedure

Two randomly paired participants were scheduled to take part in the experiment at the same time. Upon arrival, participants completed a consent form and the audiometric screening in which pitches in the range of 250–750 Hz were presented; participants’ auditory thresholds were < 30 dB SPL. The two participants were then invited to join their partner in the same testing room where they faced each other at two separate tables, each with its own force-sensitive pad setup. A screen was placed between them so that they could only see their partner’s head and shoulders, to avoid visual influence of the partners’ finger movements. Partners tapped on a force-sensitive pad using the index finger on their dominant hand. They were told that each tap would produce the next tone in the melody. Each participant was explicitly instructed to synchronize their taps with the tones they heard from their partner over headphones. The partner who produced the low-pitched melody was labeled as partner A while the one with the high-pitched melody was labeled as partner B. All participants first practiced tapping their melody (24 taps or 3 melody repetitions) before the start of the trials. Then the two partners took turns synchronizing their melody with the metronome beats.

**Figure 2:**
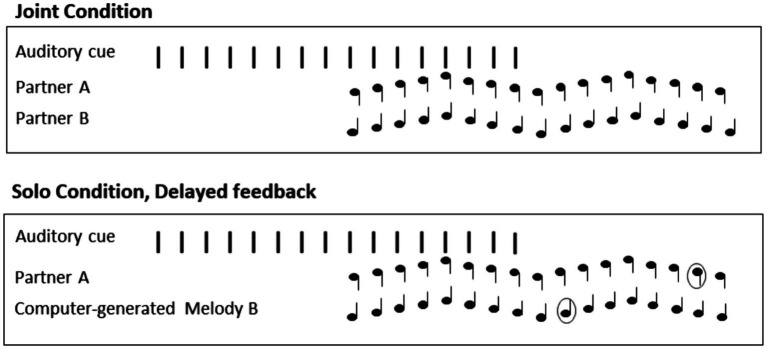
Example trials in the Joint performance and Solo-Delayed feedback conditions.

Next, the partners performed together in the Joint Performance Task (Figure 2) in which they were asked to tap together in synchrony with the metronome and to continue tapping together after the metronome ended until the sound heard over headphones ceased. After at least one practice trial and three experimental trials, the Joint performance condition was completed and participants advanced to the first Solo Intervention condition (Normal or Delayed feedback).

Each partner completed the Solo intervention condition while their partner completed musical background and Edinburgh Handedness questionnaires. Partner A was instructed to begin synchronizing their taps with a computer-generated recording of partner B’s melody after 8 beats of the metronome, and to continue synchronizing until the sound heard over headphones ceased while partner B had their headphones turned off and completed questionnaires. Then, partner B completed the same Solo intervention condition while partner A filled out their questionnaires. Neither partner could hear the other partner’s auditory feedback, which was delivered separately through headphones, during all Intervention trials.

Then participants completed the next Joint performance condition, in which they synchronized with their partner after the metronome clicks started and continued until the sound ceased, indicating the end of the trial. Then the partners completed the second Solo Intervention condition (either Normal or Delayed Intervention) and then completed the third Joint performance condition (Post-Normal or Post-Delay Joint condition). The entire experiment lasted approximately 60 min, and the participants received a small fee or course credit for their participation.

### 2.5 Data analysis

An *a priori* power analysis was conducted using G*Power 3.1.9.7 (Faul et al., 2007) to establish the minimum sample size required to test the primary hypothesis that the order of intervention conditions would influence the pairs’ joint synchronization performance (Bégel et al., 2024). Results indicated the required sample size to achieve 80% power, at a significance criterion of α = 0.05, was *N* = 25 for a two-tailed *t*-test. Thus, the obtained sample size of *N* = 25 pairs was adequate to test the study hypothesis.

Behavioral analyses were run in R Statistical Software (afex package, Singmann et al. 2023 v4.2.0–0). Tests of the synchronization measures included ANOVAs that addressed the Solo and Joint tasks separately. Measures of synchronization in the Solo tasks included signed asynchronies (stimulus onset minus participant onset), their variability (standard deviation), and their absolute value. Synchronization in the Joint tasks were measured by the standard deviation of the signed asynchronies (partner A’s onset minus partner B’s onset) and by their absolute value. Synchronization in the Solo tasks was analyzed in terms of the independent variables of Intervention condition (Delay, Normal) and Intervention Order (Delay first; Normal first), with the participant as random variable. Synchronization between partners in the Joint performance conditions were analyzed in terms of the independent variables of Post-Intervention condition (Post-Delay, Post-Normal) and Intervention Order (Delay first; Normal first), with the pair as random variable. Similar analyses were conducted on the model parameters (*k*, ω). Linear contrasts were run with the emmeans package (n 1.8.7; Lenth, 2023) and *p*-values were corrected using a Bonferroni correction.

### 2.6 Model analysis

Model analyses were conducted on the Joint performance asynchronies by taking the mean of the eight melody repetitions within each trial (*N* = 224 trials total). The delay-coupled model was fit to these asynchronies in two separate iterations, similar to previous delay-coupling applications to joint performance (Demos et al., 2019; Bégel et al., 2022). In the first stage, a global parameter search was conducted using a genetic algorithm. Parameters were allowed to vary within the following bounds: The difference between the partners’ intrinsic frequencies (𝜔1 – 𝜔2) was allowed to vary within a range of –300 ms to +300 ms, based on previously observed ranges for partners’ intrinsic frequencies (Zamm et al., 2016; Scheurich et al., 2018). The coupling strength parameters *k* were allowed to vary from 0 (no coupling) to 50. The 𝜏 parameters were allowed to vary with a range of 0 (no time delay) to 50 ms. The model was fit 10 times to each trial. In the second stage, the optimized parameter values obtained with the genetic algorithm were passed to a local search algorithm (constrained nonlinear multivariate function). Root Mean Square Error (RMSE) values for each model fit per trial were computed between the observed asynchronies and the model’s estimates of asynchronies. The best-fitting (smallest) RMSE of the 10 fits was chosen. The *tau* value associated with this best fit for each trial was then analyzed to yield the median value computed across participants and trials (*tau* = 19.7 ms), a value similar to the median *tau* obtained in other joint music performance studies (Demos et al., 2019; Bégel et al., 2022).

In a second stage, the fitting procedure was repeated with taus set to the median value (19.7 ms). Coupling and intrinsic frequency parameters were allowed to vary within identical boundaries to those used in the first model fits. The second run also consisted of 10 model fits to each trial. Fits that yielded parameter boundary cases (*k* > = 49 ms; 𝜔1 – 𝜔2 > =299 ms or <= −299 ms) were excluded from considerations of the best-fitting model, which was then chosen for each trial (based on the smallest RMSE value). The parameter values associated with the final model fits were analyzed similarly to the behavioral asynchrony measures.

## 3 Results

### 3.1 Solo intervention conditions

We conducted a two-way ANOVA on the mean intertap intervals by Solo Intervention Condition and Intervention Order, to determine whether individuals were able to maintain the cued tempo (450 ms). The analysis revealed no significant effects, indicating a stable tempo across Solo Intervention conditions with a mean intertap interval of 449.2 ms (SE = 0.179).

**Figure 3:**
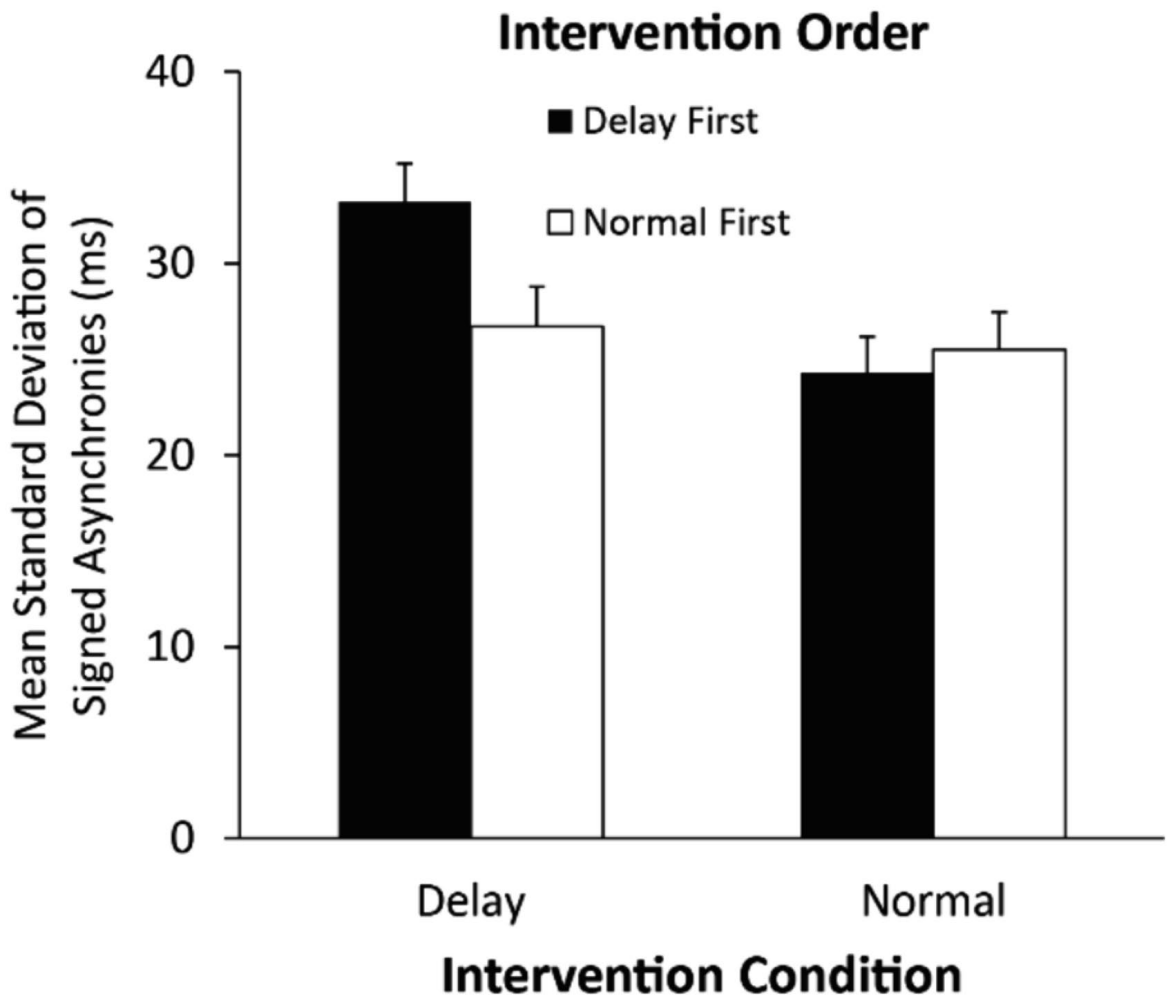
Mean standard deviations (ms) of the signed asynchronies by Solo intervention condition. Error bars indicate standard errors.

We next tested the standard deviations of the signed asynchronies in Solo interventions to determine differences due to the auditory feedback interventions. A two-way ANOVA on the standard deviations of asynchronies by Intervention condition (Delay/Normal feedback) and Intervention Order (Normal feedback first/Delayed feedback first) indicated a main effect of Intervention condition, *F*(1, 48) = 12.66, η^2^_G_ = 0.064, *p* = 0.0008, and a significant interaction of Intervention condition with Intervention Order, *F*(1, 48) = 7.42, η^2^_G_ = 0.038, *p* = 0.009. The Delayed Intervention/Delay-first order differed significantly from the Delayed Intervention/Normal-first order [linear contrasts, *t*(48) = 2.67, *p* = 0.028], the Normal Intervention condition/Delay-first order [*t*(48) = 4.53*, p* < 0.01], and the Normal Intervention/Normal-first order [*t*(48) = 2.76, *p* < 0.01]. As shown in Figure 3, greatest variability occurred during the Delayed Intervention condition when it was learned first.

**Figure 4:**
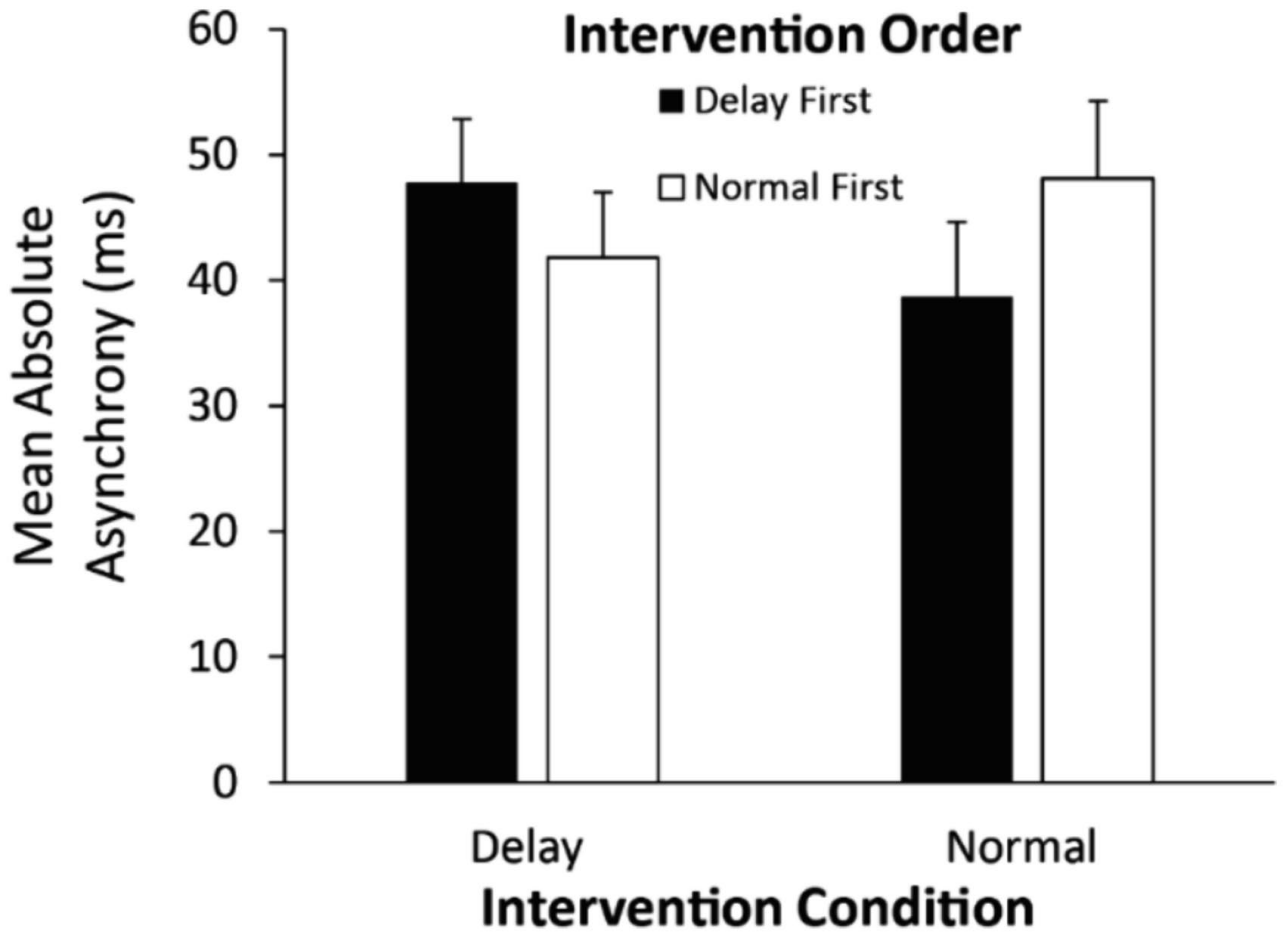
Mean absolute asynchronies (ms) in the Solo Intervention conditions by Intervention Condition and Intervention Order. Error bars indicate standard errors.

We also tested the absolute asynchronies between individuals’ taps and the computer-generated melody in the Solo Intervention conditions. A two-way ANOVA on the mean absolute asynchronies by Intervention Condition (Delay/Normal feedback) and Intervention Order (Normal feedback first/Delayed feedback first) revealed a significant interaction [*F*(1, 48) = 6.88, η^2^_G_ = 0.019, *p* = 0.011]. As shown in Figure 4, the absolute asynchronies were greater in the intervention that was conducted first, suggesting some improvement over the course of the experiment. There were no significant main effects.

Finally, we computed the signed asynchronies in the Solo intervention conditions in order to determine whether individuals anticipated the computer-generated recording. Overall, the asynchronies were negative in each condition, indicating that the participants anticipated the metronome cue (mean = −39.1 ms). The same ANOVA on the signed asynchronies by Intervention condition and Intervention Order showed no main effects but a statistically significant interaction [*F*(1, 48) = 5.09; η^2^_G_ = 0.014, *p* = 0.03]. Performance during the Solo intervention conditions was more anticipatory during the Normal feedback intervention than during the Delayed feedback intervention, when the participants received the Normal feedback intervention first (Bonferroni-corrected contrasts, *t* = 2.86, *p* < 0.04). Asynchronies were equivalent in Delayed and Normal feedback conditions when the Delayed feedback condition was first.

### 3.2 Joint synchronization conditions

**Figure 5:**
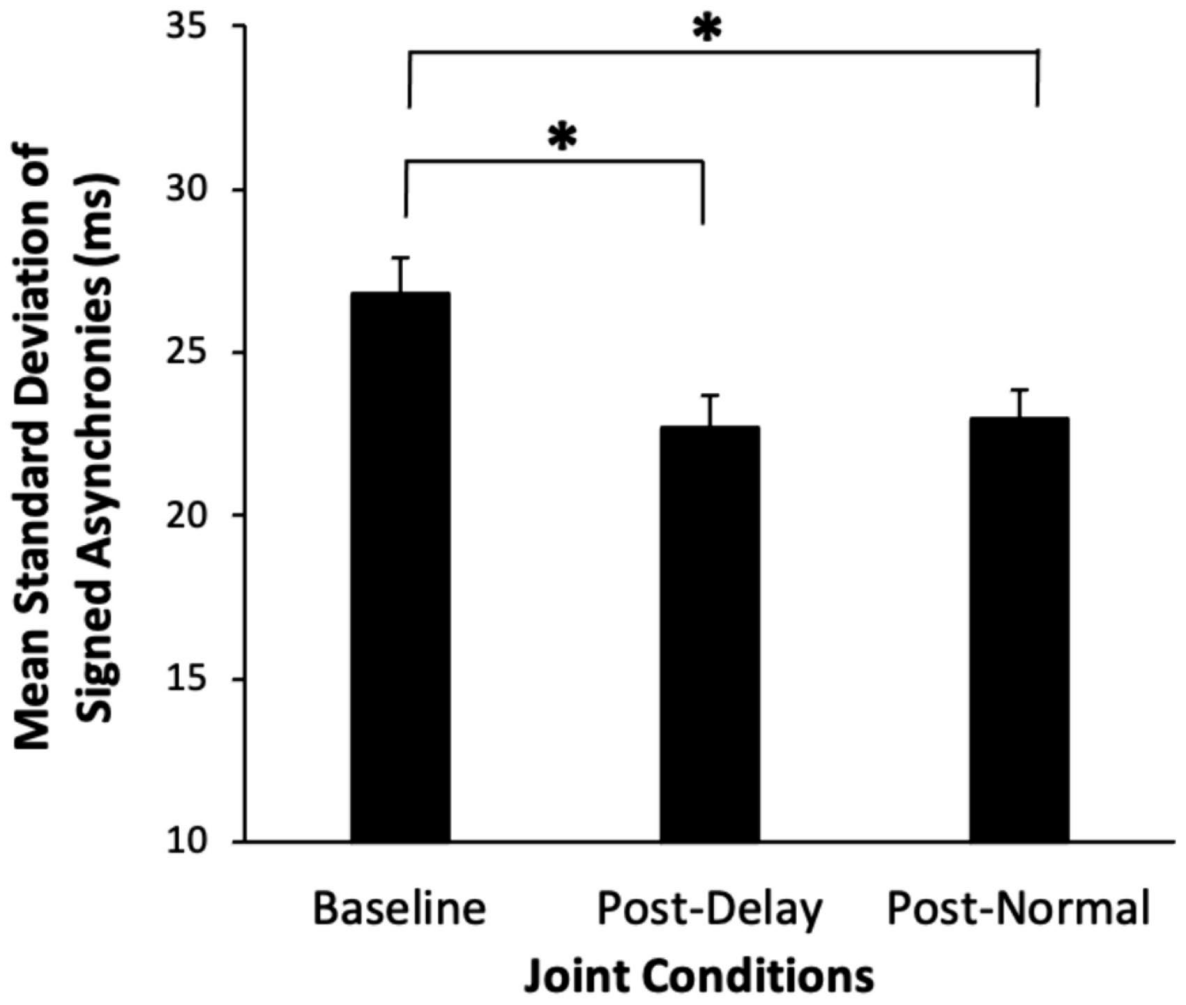
Mean standard deviations of signed asynchronies (ms) in the three Joint performance conditions. Error bars indicate standard errors. Asterisks indicate significant differences (*p* < 0.05).

We first compared the Baseline Joint performance condition (before Solo Interventions) with the synchrony observed in the other Joint performance conditions. An ANOVA on the standard deviations of the asynchronies by Joint condition (Baseline, Post-Delay intervention, Post-Normal intervention) and Intervention Order (Delay first/Normal first) indicated a significant main effect of Joint condition [*F*(2, 46) = 26.8, η^2^_G_ = 0.136, *p* < 0.00001]. As shown in Figure 5, the standard deviations were larger in the Baseline condition (mean SD = 26.8 ms) than in the Post-Delay condition [mean SD = 22.7 ms, Bonferroni-corrected *t*(23) = 5.48] and the Post-Normal condition [mean SD = 23.0; Bonferroni-corrected *t*(23) = 6.24]. Thus, participants showed improvement in synchronization from Baseline to later Joint performances, and the pairs that were assigned to the different Solo Intervention orders did not differ at Baseline.

Next, we examined the mean absolute asynchronies by Joint Condition (Baseline, Post-Delay, Post-Normal) and Intervention order (Delay first/Normal first). This ANOVA yielded a non-significant effect of Post-Intervention condition [*F*(2, 46) = 2.74, *p* = 0.075] with similar patterns to the standard deviations; absolute asynchronies tended to be higher in the Baseline condition (mean SD = 25.4 ms) than in the Post-Delay (mean SD = 23.0 ms) or Post-Normal conditions (mean SD = 22.9 ms). No other tests approached significance.

**Figure 6:**
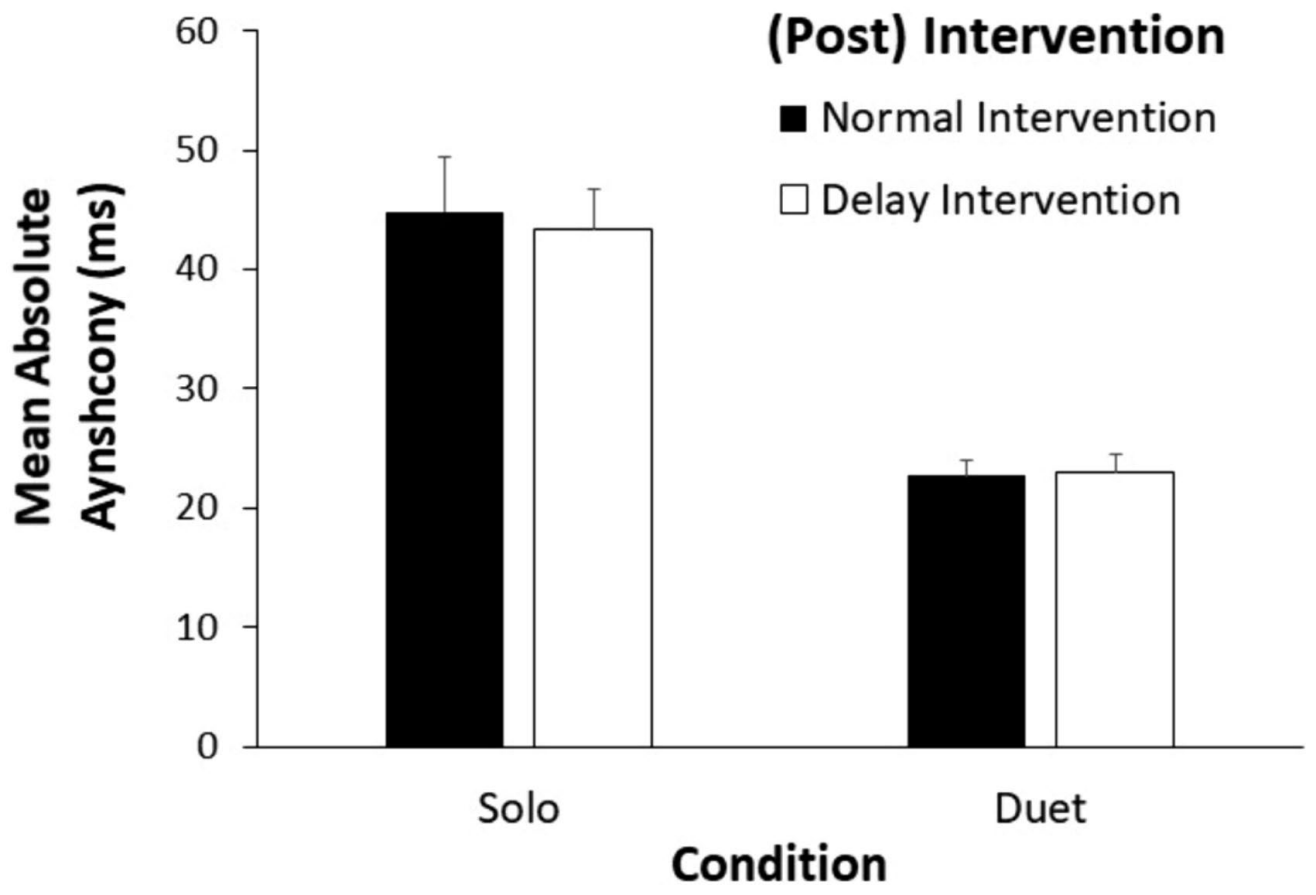
Mean absolute asynchronies (ms) by Condition (Solo Intervention, Joint Synchrony) and by Solo Intervention Order. Error bars indicate standard errors.

Finally, we compared the absolute asynchronies across the Solo intervention conditions and the Joint performance conditions, to test whether performing with a computer-generated performance (that permitted only unidirectional coupling from human to computer) or with a slightly irregular partner (that permitted bidirectional coupling from human to human) would generate better synchrony. A two-way ANOVA on each pair’s absolute asynchronies by Task (Solo/Joint) and Intervention type (Delay/Post-Delay or Normal/Post-Normal) showed a significant main effect of Task [*F*(1, 24) = 61.70, η^2^_G_ = 0.340, *p* < 0.00001] and no other significant main effects or interactions. As shown in Figure 6, participants were more synchronous when performing with a partner than when performing with a temporally regular recording – whether or not that recording contained occasional delayed auditory feedback.

### 3.3 Model fits

**Figure 7:**
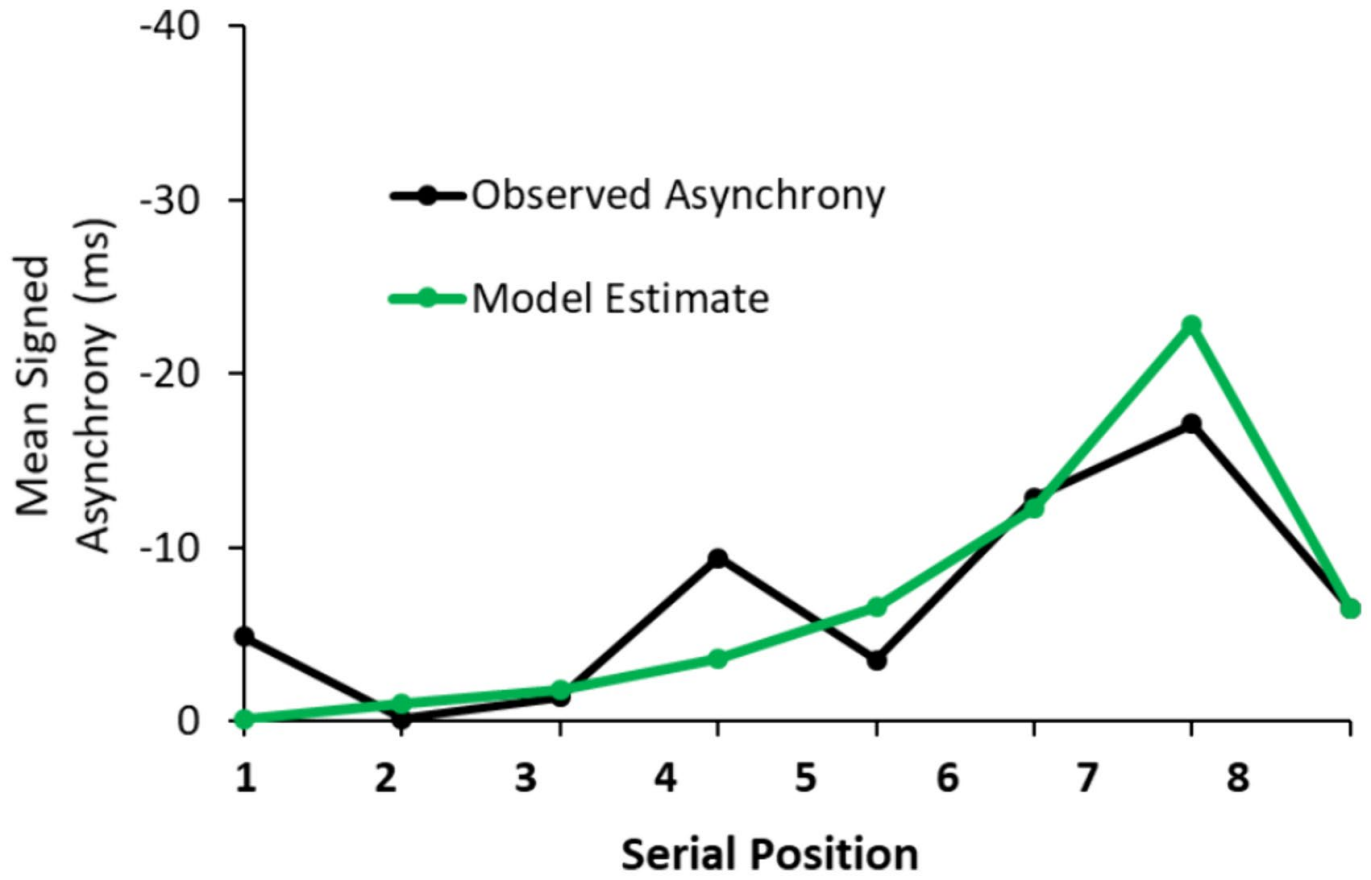
A sample trial from Joint Synchronization condition following the Solo - Normal feedback Intervention with observed signed asynchronies (Partner 1 – Partner 2, ms) and delay-coupled model fits.

We examined the model fits to the partners’ mean signed asynchronies in the Joint performance conditions. An example of the model’s predicted asynchrony values with the observed asynchrony values from one pair’s performance is shown in Figure 7 for the Joint performance condition that followed the Solo Normal-feedback Intervention.

**Figure 8:**
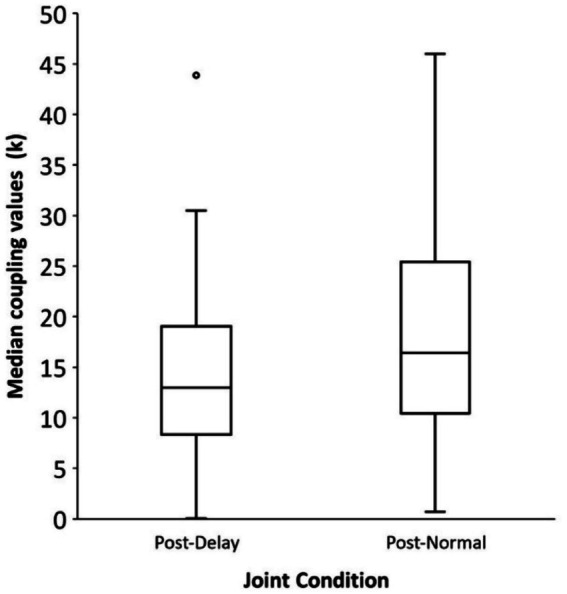
Median coupling values (k) from the delay-coupling model fits by Joint Synchronization condition. Box edges indicate 25th to 75th percentiles; error bars indicate 5th to 95th percentiles.

Statistical analyses were performed on the coupling parameter *k* for each partner, to compare the effects of Intervention conditions on the pairs’ Joint performance. A Wilcoxon signed-ranks test on the median coupling parameter values in each condition indicated that the coupling values were significantly higher in the Post-Normal Joint performance condition (Mdn = 16.44) than in the Post-Delay Joint performance condition (Mdn = 12.96, *z* = 1.97, *p* = 0.024). As shown in Figure 8, the model’s coupling values were increased in the joint synchronization experienced after the Normal feedback intervention relative to the Delayed feedback intervention. Decreased coupling during synchronization with a partner that followed the Delayed feedback Intervention suggests that the Delayed feedback disrupted participants’ subsequent ability to synchronize their productions.

Next, we tested the delay-coupled model fits to the intrinsic frequency (ω) parameters. Intrinsic frequency differences between partners are related to tempo or rate of performance; a larger difference between partners, computed in absolute ms, is expected to yield larger mean absolute asynchronies. A Wilcoxon signed-ranks test on the absolute value of each pair’s ω differences by Joint performance condition (Post-normal, Post-delay) indicated no significant differences (*z* = 0.38, *p* = 0.35). Therefore, it is unlikely that the intrinsic frequency parameter accounted for the Solo Intervention effects on the coupling parameters in subsequent Joint synchronization.

Finally, we compared the delay-coupling model with a simpler model that removed the coupling parameter, to ensure its necessity in accounting for Joint synchronization performance. The bidirectional delay-coupling model was compared with a linear model that contained only the intrinsic frequency (ω) parameter while k, the coupling term, was set to 0 (thus cancelling the time delay parameter as well; see Equation 1). Root mean squared error values (RMSE) were generated for the two models fitted to the partners’ asynchronies in the Joint performance conditions. A one-way ANOVA was then conducted on the RMSE values by model (delay-coupling model and intrinsic frequency model). The RMSE values indicated a statistically significant difference among the models [*F*(1,24) = 49.157, η^2^_G_ = 0.131, *p* < 0.001]. The model fits with the coupling value set to 0 produced higher average RMSE values (*M* = 7.348), indicating a worse fit, than the model fits with the coupling term allowed to vary (*M* = 6.359). Thus, the coupling parameter was necessary to capture the partners’ Joint performance synchronization.

## 4 Discussion

The impact of individual synchronization practice on joint synchronization was examined with musically trained partners in a within-pair design that presented different individual (solo) practice conditions. Effects of Solo synchronization practice with normal or delayed auditory feedback were evaluated on subsequent Joint duet performance as participants synchronized the production of simple melodies with an auditory cue (Solo conditions) or with a partner (Joint conditions). The within-pair design allowed us to compare Intervention order effects on the partners’ subsequent joint synchronization. The application of a delay-coupling model to the partners’ joint synchronization measures allowed us to compare the amount of coupling between partners as a function of the individual practice conditions.

The auditory feedback manipulations in the Solo intervention conditions resulted in higher variability in asynchronies during the Delayed Feedback condition than the Normal feedback condition. These results are consistent with previous findings that indicate that delayed auditory feedback hinders participants’ attempts to synchronize (Repp et al., 2011; Bégel et al., 2022). In addition, absolute asynchronies in the Solo intervention conditions indicated that the auditory feedback type interacted with the order of Intervention conditions; participants were worse at synchronization in the first Solo intervention they experienced, and better at synchronization by the last Solo intervention, suggesting that learning occurred despite the type of auditory feedback.

The dyads’ Joint synchronization performance indicated that the asynchrony variability improved from the initial Joint performance to later Joint performance, also supporting a learning effect as participants progressed through the tasks. Fortunately, there were no initial differences in Joint synchrony across the groups of participants who completed different two orders of the Solo interventions, suggesting that pairwise differences in synchronization did not account for the Solo intervention effects. Although the Solo intervention conditions did not directly influence the immediately following Joint performance asynchronies, the model’s coupling parameter fits suggested that the auditory feedback manipulations in the Solo practice influenced bidirectional synchronization in the subsequent Joint performances.

Fits of the delay-coupling model to the Joint synchronization performances indicated increased coupling values between partners when the Joint performances followed the normal auditory feedback (Solo) practice, relative to the delayed feedback practice. Furthermore, the remaining model parameter that was allowed to vary, the intrinsic frequency parameter, did not differ across the Joint synchronization conditions. We also tested whether the coupling parameter was necessary by comparing the delay-coupling model fits to those of a reduced model that contained only the intrinsic frequency parameters (with coupling set to 0). The reduced model provided a substantially poorer fit, suggesting that the coupling parameter was necessary to account for the partners’ asynchronies in joint performance. Future work may compare the delay-coupled model presented here with other numerical solutions (*cf.* Roman et al., 2019; Shahal et al., 2020; Calabrese et al., 2022), as well as with analytical solutions such as linear delay differential equations (*cf.* Yi and Ulsoy, 2006) and networks of delay-coupled oscillators (Pérez et al., 2011).

Finally, musicians’ synchronization was compared across Solo performances and Joint (dyadic) performance. The absolute asynchronies were greater in the Solo performances with a computer-generated performance than in the Joint performances with a partner. This finding may seem surprising, as the Solo interventions contained temporally regular inter-tone intervals (with the exception of 25% of delayed tone onsets in one condition), whereas all of the Joint synchronizations contained partners’ normal temporal variability. These findings can be reconciled if partners’ Joint synchronization reflects *predictable* temporal variability that allows each performer to adapt to their partner; previous studies have also documented reduced asynchronies for duet partners compared with the same partner’s synchronization with a temporally regular recording (Demos et al., 2017). The greater predictability of duet synchronization is also consistent with findings that showed better fit of nonlinear oscillators to the normal temporal variability in piano performances than to metronomically regular performances (Large and Palmer, 2002). Future studies may address how bidirectional coupling between partners develops as they learn to synchronize with more complex music than the simple melodies used here.

The current study addressed musical synchronization in the context of Western classical music forms. Some studies have identified different performance timing in other genres. Butterfield (2010) examined asynchronous timing between bass and drums players in swing groove music, in which a sense of constant time was negotiated between performers. Johansson’s (2010) analysis of Scandinavian folk music for fiddle similarly argued that rhythmic tolerance calls for tone onset ambiguities among performers. Danielsen (2018) identified extended beats in a musical genre of neo-soul groove, where an expectation for active anticipation among partners can yield aesthetic choices that result in asynchronies. Thus, performance norms in groove, jazz, and neo-soul musical forms may differ from classical music performance norms typical of the current study, which included an emphasis on temporal precision and synchronous tones.

In conclusion, the findings document short-term learning effects of solo performance on joint synchronization by musically trained partners. The Joint synchronization conditions indicated that synchronization with a partner becomes more accurate over the course of the experiment; the Solo Intervention conditions demonstrated that the quality of auditory feedback influences coupling between duet partners in future joint performances. This finding reinforces the validity of musicians’ common solo practice methods, which can enhance subsequent performance in ensemble situations. Future directions may address distinctions between unidirectional coupling of individual practice with a recording (common in musicians’ play-along practice albums) and bidirectional coupling that arises in joint performance. Physiological changes that occur during ensemble performance conditions, such as joint influences on respiration or heart rate, may also impact partners’ coupling (Wright et al., 2022; Høffding et al., 2023). Finally, further investigations may compare influences of solo practice on joint synchronization in terms of frequency of practice (such as performers who practice music daily and those who do not).

## Data availability statement

The datasets presented in this study can be found in online repositories. The names of the repository/repositories and accession number(s) can be found below: https://osf.io/t72ze/?view_only=87d29c4afcb64e6b889177802554f2a2.

## Ethics statement

The studies involving humans were approved by McGill University Research Ethics Board. The studies were conducted in accordance with the local legislation and institutional requirements. The participants provided their written informed consent to participate in this study.

## Author contributions

PP: Data curation, Formal analysis, Methodology, Writing – original draft, Writing – review & editing. VB: Conceptualization, Formal analysis, Writing – original draft, Writing – review & editing. CP: Conceptualization, Formal analysis, Funding acquisition, Methodology, Project administration, Resources, Supervision, Writing – original draft, Writing – review & editing.

## References

[ref1] Aschersleben G. Prinz W. (1995). Synchronizing actions with events: the role of sensory information. Percept. Psychophys. 57, 305–317. doi: 10.3758/BF03213056, PMID: 7770322

[ref2] Bégel V. Demos A. P. Palmer C. (2024). Duet synchronization interventions affect social interactions. Sci. Rep 14:9930. doi: 10.1038/s41598-024-60485-w, PMID: 38688922 PMC11061167

[ref3] Bégel V. Demos A. P. Wang M. Palmer C. (2022). Social interaction and rate effects in models of musical synchronization. Front. Psychol. 13:865536. doi: 10.3389/fpsyg.2022.865536, PMID: 35783789 PMC9242395

[ref4] Bishop L. Bailes F. Dean R. T. (2014). Performing musical dynamics: how crucial are musical imagery and auditory feedback for expert and novice musicians? Music. Percept. 32, 51–66. doi: 10.1525/mp.2014.32.1.51

[ref5] Butterfield M. (2010). Participatory discrepancies and the perception of beats in jazz. Music. Percept. 27, 157–176. doi: 10.1525/mp.2010.27.3.157

[ref6] Calabrese C. Bardy B. G. De Lellis P. Di Bernardo M. (2022). Modeling frequency reduction in human groups performing a joint oscillatory task. Front. Psychol. 12:753758. doi: 10.3389/fpsyg.2021.753758, PMID: 35058838 PMC8765722

[ref7] Caramiaux B. Bevilacqua F. Wanderley M. M. Palmer C. (2018). Dissociable effects of practice variability on learning motor and timing skills. PLoS One 13:e0193580. doi: 10.1371/journal.pone.0193580, PMID: 29494670 PMC5832267

[ref8] Castro-Meneses L. J. Sowman P. F. (2018). Stop signals delay synchrony more for finger tapping than vocalization: a dual modality study of rhythmic synchronization in the stop signal task. Peer J. 6:e5242. doi: 10.7717/peerj.5242, PMID: 30013856 PMC6046193

[ref9] Clark A. (1997). Being there: putting brain, body and world together again. Philos. Rev. 107:647,

[ref10] Danielsen A. (2018). “Pulse as dynamic attending: analysing beat bin metre in neo soul grooves” in The Routledge companion to popular music analysis: expanding approaches. eds. Scotto C. Smith K. M. Brackett J. (New York: Routledge), 179–188.

[ref11] Delius J. A. M. Mueller V. (2022). Interpersonal synchrony when singing in a choir. Front. Psychol. 13:1087517. doi: 10.3389/fpsyg.2022.1087517 36710769 PMC9875726

[ref12] Demos A. P. Carter D. J. Wanderley M. M. Palmer C. (2017). The unresponsive partner: roles of social status, auditory feedback, and animacy in coordination of joint music performance. Front. Psychol. 8:149. doi: 10.3389/fpsyg.2017.00149, PMID: 28261123 PMC5306131

[ref13] Demos A. P. Layeghi H. Wanderley M. M. Palmer C. (2019). Staying together: a bidirectional delay–coupled approach to joint action. Cogn. Sci. 43:e12766. doi: 10.1111/cogs.12766, PMID: 31446664

[ref14] Dumas G. Fairhurst M. T. (2021). Reciprocity and alignment: quantifying coupling in dynamic interactions. R. Soc. Open Sci. 8:210138. doi: 10.1098/rsos.210138, PMID: 34040790 PMC8113897

[ref15] Faul F. Erdfelder E. Lang A.-G. Buchner A. (2007). G*power 3: a flexible statistical power analysis program for the social, behavioral, and biomedical sciences. Behav. Res. Methods 39, 175–191. doi: 10.3758/BF03193146, PMID: 17695343

[ref16] Finney S. A. (1997). Auditory feedback and musical keyboard performance. Music. Percept. 15, 153–174. doi: 10.2307/40285747

[ref17] Finney S. A. (2001). FTAP: a Linux-based program for tapping and music experiments. Behav. Res. Methods Instrum. Comput. 33, 65–72. doi: 10.3758/BF03195348, PMID: 11296721

[ref18] Finney S. Palmer C. (2003). Auditory feedback and memory for music performance: sound evidence for an encoding effect. Mem. Cogn. 31, 51–64. doi: 10.3758/BF03196082, PMID: 12699143

[ref19] Highben Z. Palmer C. (2004). Effects of auditory and motor mental practice in memorized piano performance. Bull. Counc. Res. Music. Educ. 159, 58–65.

[ref20] Høffding S. Yi W. Lippert E. Sanchez V. G. Bishop L. Laeng B.. (2023). Into the hive-mind: shared absorption and cardiac interrelations in expert and student string quartets. Music Sci. 6:205920432311685. doi: 10.1177/20592043231168597

[ref21] Jacoby N. Tishby N. Repp B. H. Ahissar M. Keller P. E. (2015). Parameter estimation of linear sensorimotor synchronization models: phase correction, period correction, and ensemble synchronization. Timing Time Percept. 3, 52–87. doi: 10.1163/22134468-00002048

[ref22] Johansson M. (2010). “The concept of rhythmic tolerance: examining flexible grooves in Scandinavian folk fiddling” in Musical rhythm in the age of digital reproduction. ed. Danielsen A. (Franham, UK: Ahsgate), 69–83.

[ref23] Keller P. E. Knoblich G. Repp B. H. (2007). Pianists duet better when they play with themselves: on the possible role of action simulation in synchronization. Conscious. Cogn. 16, 102–111. doi: 10.1016/j.concog.2005.12.004, PMID: 16466932

[ref24] Keller P. E. Novembre G. Loehr J. (2016). “Musical ensemble performance: representing self, other and joint action outcomes” in Shared representations: sensorimotor foundations of social life. eds. Obhi S. S. Cross E. S. (Cambridge: Cambridge University Press), 280–310.

[ref25] Large E. W. Herrera J. Velasco M. J. (2015). Neural networks for beat perception in musical rhythm. Front. Syst. Neurosci. 9:159. doi: 10.3389/fnsys.2015.00159, PMID: 26635549 PMC4658578

[ref26] Large E. W. Palmer C. (2002). Perceiving temporal regularity in music. Cogn. Sci. 26, 1–37. doi: 10.1207/s15516709cog2601_1

[ref27] Lenth R. V. (2023). Estimated marginal means, aka least-squares means [R package emmeans version 1.8.9]: The Comprehensive R Archive Network. Available at: https://github.com/rvlenth/emmeans

[ref28] Liebermann-Jordanidis H. Novembre G. Koch I. Keller P. E. (2021). Simultaneous self-other integration and segregation support real-time interpersonal coordination in a musical joint action task. Acta Psychol. 218:103348. doi: 10.1016/j.actpsy.2021.103348, PMID: 34058671

[ref29] Luciani M. G. Cortelazzo A. Proverbio A. M. (2022). The role of auditory feedback in the motor learning of music in experienced and novice performers. Sci. Rep. 12:19822. doi: 10.1038/s41598-022-24262-x, PMID: 36396694 PMC9671877

[ref30] Machado J. N. Matias F. S. (2020). Phase bistability between anticipated and delayed synchronization in neuronal populations. Phys. Rev. E 102:032412. doi: 10.1103/PhysRevE.102.032412, PMID: 33075861

[ref31] Mathias B. M. Palmer C. Perrin F. Tillmann B. (2015). Sensorimotor learning enhances expectations during auditory perception. Cereb. Cortex 25, 2238–2254. doi: 10.1093/cercor/bhu030, PMID: 24621528

[ref32] Moiseff A. Copeland J. (2010). Firefly synchrony: a behavioral strategy to minimize visual clutter. Science 329:181. doi: 10.1126/science.1190421, PMID: 20616271

[ref33] Palmer C. (2005). Sequence memory in music performance. Curr. Dir. Psychol. Sci. 14, 247–250. doi: 10.1111/j.0963-7214.2005.00374.x

[ref34] Palmer C. Demos A. P. (2022). Are we in time? How predictive coding and dynamical systems explain musical synchrony. Curr. Dir. Psychol. Sci. 31, 147–153. doi: 10.1177/09637214211053635, PMID: 35400858 PMC8988459

[ref35] Pérez T. Eguíluz V. M. Arenas A. (2011). Phase clustering in complex networks of delay-coupled oscillators. Chaos 21:025111. doi: 10.1063/1.3595601, PMID: 21721789

[ref36] Repp B. H. (1999). Effects of auditory feedback deprivation on expressive piano performance. Music. Percept. 16, 409–438. doi: 10.2307/40285802

[ref37] Repp B. H. London J. Keller P. E. (2011). Perception–production relationships and phase correction in synchronization with two-interval rhythms. Psychol. Res. 75, 227–242. doi: 10.1007/s00426-010-0301-8, PMID: 20644955

[ref38] Repp B. H. Su Y.-H. (2013). Sensorimotor synchronization: a review of recent research (2006–2012). Psychon. Bull. Rev. 20, 403–452. doi: 10.3758/s13423-012-0371-2, PMID: 23397235

[ref39] Roman I. R. Washburn A. Large E. W. Chafe C. Fujioka T. (2019). Delayed feedback embedded in perception-action coordination cycles results in anticipation behavior during synchronized rhythmic action: a dynamical systems approach. PLoS Comput. Biol. 15:e1007371. doi: 10.1371/journal.pcbi.1007371, PMID: 31671096 PMC6822724

[ref40] Scheurich R. Zamm A. Palmer C. (2018). Tapping into rate flexibility: musical training facilitates synchronization around spontaneous production rates. Front. Psychol. 9:458. doi: 10.3389/fpsyg.2018.00458, PMID: 29681872 PMC5897499

[ref41] Shahal S. Wurzberg A. Sibony I. Duadi H. Shniderman E. Weymouth D.. (2020). Synchronization of complex human networks. Nat. Commun. 11:3854. doi: 10.1038/s41467-020-17540-7, PMID: 32782263 PMC7419301

[ref42] Singmann H. Bolker B. Westfall J. Aust F. Ben-Shachar M. S. (2023). Analysis of factorial experiments [R package AFEX version 1.3-0].: The Comprehensive R Archive Network.

[ref43] Stambaugh L. A. (2011). When repetition isn’t the best practice strategy: effects of blocked and random practice schedules. J. Res. Music. Educ. 58, 368–383. doi: 10.1177/0022429410385945

[ref44] Stepp N. (2009). Anticipation in feedback-delayed manual tracking of a chaotic oscillator. Exp. Brain Res. 198, 521–525. doi: 10.1007/s00221-009-1940-0, PMID: 19609514 PMC3724402

[ref45] Stepp N. Turvey M. T. (2010). On strong anticipation. Cogn. Syst. Res. 11, 148–164. doi: 10.1016/j.cogsys.2009.03.003, PMID: 20191086 PMC2827858

[ref46] Strogatz S. H. Stewart I. (1993). Coupled oscillators and biological synchronization. Sci. Am. 269, 102–109. doi: 10.1038/scientificamerican1293-102 8266056

[ref47] Tranchant P. Scholler E. Palmer C. (2022). Endogenous rhythms influence musicians’ and non-musicians’ interpersonal synchrony. Sci. Rep. 12:12973. doi: 10.1038/s41598-022-16686-2, PMID: 35902677 PMC9334298

[ref48] Van Der Steen M. C. Keller P. E. (2013). The adaptation and anticipation model (ADAM) of sensorimotor synchronization. Front. Hum. Neurosci. 7. doi: 10.3389/fnhum.2013.00253, PMID: 23772211 PMC3677131

[ref49] Voss H. U. (2000). Anticipating chaotic synchronization. Phys. Rev. 61, 5115–5119, 10.1103/physreve.61.5115 11031554

[ref50] Wing A. M. Endo S. Bradbury A. Vorberg D. (2014). Optimal feedback correction in string quartet synchronization. J. R. Soc. Interface 11:20131125. doi: 10.1098/rsif.2013.1125, PMID: 24478285 PMC3928944

[ref51] Wright S. E. Bégel V. Palmer C. (2022). Physiological influences of music in perception and action (elements in perception). Cambridge: Cambridge University Press.

[ref52] Yi S. Ulsoy A. G. (2006). Solution of a system of linear delay differential equations using the matrix Lambert function. Proceedings of the American Control Conference

[ref53] Zamm A. Wellman C. Palmer C. (2016). Endogenous rhythms influence interpersonal synchrony. J. Exp. Psychol. Hum. Percept. Perform. 42, 611–616. doi: 10.1037/xhp0000201, PMID: 26820249

